# Cancer survival in Kampala, Uganda

**DOI:** 10.1038/sj.bjc.6602540

**Published:** 2005-04-12

**Authors:** A Gondos, H Brenner, H Wabinga, D M Parkin

**Affiliations:** 1 Department of Epidemiology, German Centre for Research on Ageing, Berghiemer Str. 20, 69115 Heidelberg, Germany; 2 Kampala Cancer Registry, Department of Pathology, Makerere University, PO BOX, 7072, Kampala, Uganda; 3 Unit of Descriptive Epidemiology, International Agency for Research on Cancer, 69372 Lyon, France

**Keywords:** cancer survival, Uganda, Africa, population-based survival

## Abstract

Epidemiological data on the occurrence of cancer in sub-Saharan Africa are sparse, and population-based cancer survival data are even more difficult to obtain due to various logistic difficulties. The population-based Cancer Registry of Kampala, Uganda, has followed up the vital status of all registered cancer patients with one of the 14 most common forms of cancer, who were diagnosed and registered between 1993 and 1997 in the study area. We report 5-year absolute and relative survival estimates of the Ugandan patients and compare them with those of black American patients diagnosed in the same years and included in the SEER Program of the United States. In general, the prognosis of cancer patients in Uganda was very poor. Differences in survival between the two patient populations were particularly dramatic for those cancer types for which early diagnosis and effective treatment is possible. For example, 5-year relative survival was as low as 8.3% for colorectal cancer and 17.7% for cervical cancer in Uganda, compared with 54.2 and 63.9%, respectively, for black American patients. The collection of good-quality follow-up data was possible in the African environment. The very poor prognosis of Ugandan patients is most likely explained by the lack of access to early diagnosis and treatment options in the country. On the policy level, the results underscore the importance of the consistent application of the national cancer control programme guidelines as outlined by the World Health Organization.

Although cancer registries have been present in sub-Saharan Africa for 50 years, they are few, and population-based epidemiological cancer data are sparse. From the region, population-based cancer survival estimates for many different cancers were only reported once before, and two further studies restricted to cervical cancer already make the list of population-based cancer survival reports complete (Wabinga *et al* 2003; Chokunonga *et al*, 2004; Gondos *et al*, 2004). This paper provides survival estimates of patients diagnosed with one of the 14 most common cancers in Kampala, Uganda, 1993–1997. We present 5-year absolute and relative survival estimates, and we compare the results with those of black American cancer patients diagnosed in the same period and registered in the Surveillance, Epidemiology and End Results (SEER) programme of the National Cancer Institute of the United States (SEER, 2002).

## METHODS

### Database

This study is based on data of the Kampala Cancer Registry (Kampala, Uganda), which was established in 1951 and is one of the longest standing cancer registries on the African continent. The registry covers the area of Kyadondo county, which comprises Kampala, the capital city of Uganda and neighbouring urban and semiurban areas, with an estimated total population of 1.2 million (1998). Although registry operations were massively influenced by the political turmoil after 1971 and were actually stopped between 1980 and 1989, the functioning has been undisturbed since the reopening of registration in 1989.

Cancer incidence data from the registry have been published in detail, describing the changes across several decades and into the era of AIDS (Wabinga *et al*, 1993, 2000; Parkin *et al*, 1999). In the 1990s, the cancers with the highest incidence were Kaposi sarcoma, prostate cancer, and oesophageal cancer among men and cervical cancer, breast cancer, and Kaposi sarcoma among women (Wabinga *et al*, 2000). With the AIDS epidemic, large increases in the incidence of Kaposi sarcoma, squamous cell carcinoma of the conjunctiva, and non-Hodgkin's lymphoma have been observed (Parkin *et al*, 1999). The registry is one of the African registries taking part in an international effort to assess cancer occurrence and management in developing countries. The project is coordinated by the International Agency of Research on Cancer (IARC, Lyons/France).

The registry methods and results have been described previously (Wabinga *et al*, 1993, 2000). Case finding is conducted by active search for newly diagnosed cases identified from the records of three pathology laboratories and six hospitals in the area and from the Uganda Hospice. Death certificates are rarely completed for deaths occurring outside hospital, and are therefore not used as a source of information. The completeness of registration was assessed during the mid-1990s and was found to be around 90% for incident cases (Parkin *et al*, 2001).

This study includes all patients with one of the 13 most common cancers reported to the Kampala Cancer Registry between 1993 and 1997, except for Kaposi sarcoma (in total 1376 patients between 1993 and 1997), of whom only patients with a known HIV status were included (*N*=249). Most of these patients participated in a recent case–control study of HIV and cancer in the area (Ziegler *et al*, 1997).

The follow-up was carried out from January 2001 to June 2002, the effective cutoff date for the follow-up of patients' vital status was 31 December 1999. The process of follow-up posed a serious challenge to the registry, as medical or civil death certification is not performed routinely in the country (Wabinga *et al*, 2003). Therefore, if institutional records did not provide sufficient information on the vital status of the patient, home visits were undertaken. These were often extremely difficult to carry out, as street addresses are not used, and residence is defined simply as the village or city area. Consequently, despite enormous efforts by the registry, many patients could not be fully traced for the entire follow-up period. These patients with incomplete follow-up were included in the analysis, and were censored alive in the month in which they were last seen.

### Statistical analysis

For each cancer site, absolute (observed) and relative survival estimates were calculated. Relative survival figures quantify net (cancer-related) survival, and they are obtained as the ratios of absolute (observed) survival and the expected survival in the absence of cancer (Ederer *et al*, 1961). The latter was determined from age and sex-specific WHO life table estimates for Uganda, which were calculated for the year 2000 and are based on approximated model calculations, since vital statistics are not available (Lopez *et al*, 2001). These population life tables represent the survival experience of the entire Ugandan population and take the AIDS epidemic into account. Calculations of relative survival were performed according to Hakulinen's method (Hakulinen, 1982), using the SAS macro *periodh* (Brenner *et al*, 2002). Standard errors of survival estimates were calculated according to Greenwood's (1926) method.

Absolute and relative 5-year survival estimates for Ugandan patients were compared with the corresponding estimates for black patients diagnosed in the same years in the United States. For the black American patients, data from the cancer registries included in the SEER programme were used, a data source renowned for its high quality in all aspects of cancer registration. Owing to the large age differences between the Ugandan and the SEER cancer populations, the estimates for black American patients were adjusted to the age distributions of the Ugandan patients using three broad age groups (<30, 30–59, and ⩾60). The adjustment of relative survival estimates was performed as described by Brenner and Hakulinen (2003).

## RESULTS

Overall, 2337 patients were diagnosed with cancer and reported to the Kampala cancer registry between 1993 and 1997. Out of these, 506 (21.7%) patients were excluded from the analysis because there was no information on their vital status after diagnosis or they had other critical data missing that was required for the analysis. Table 1 describes the cancer patient population included in the survival analysis. Among patients who were included in the analysis, 26.8% were lost at some point during the follow-up and could not be fully traced until death or the cutoff date. The proportion of histologically verified cases was 61.8% overall, with a maximum of 83.2% for patients with Kaposi sarcoma, and a minimum of 33.3% for patients with liver cancer.

As described in Table 2, the median age of patients was much lower in Uganda than in the United States for almost all forms of cancers. The only exceptions were patients with prostate cancer and thyroid cancer, who had similar median ages. Data of patients with eye cancer are not comparable, as almost all black American patients included in the data set had retinoblastoma, a childhood cancer, whereas the majority of Ugandan patients were suffering from squamous cell carcinoma of the conjunctiva. We provide separate median ages for HIV-positive and HIV-negative Ugandan Kaposi sarcoma patients. As a result of the difference in the nature of eye cancers present in the Ugandan and American populations and the lack of information on HIV status of American patients with Kaposi sarcoma, no comparative analysis is provided for these two cancers.

Table 3 presents the absolute and relative survival estimates for the Ugandan cancer patient populations. The prognosis of Ugandan cancer patients was generally very poor. The lowest survival was recorded for patients with nasopharyngeal, stomach ,or lung cancer, none of whom survived 5 years. Patients with other gastrointestinal tract cancers also had very low survival. The highest survival figures were seen for HIV-negative Kaposi sarcoma patients, and for patients with breast cancer or prostate cancer: the 5-year relative survival of these patients was 65.7, 45.4, and 46.9%, respectively.

**Figure 1:**
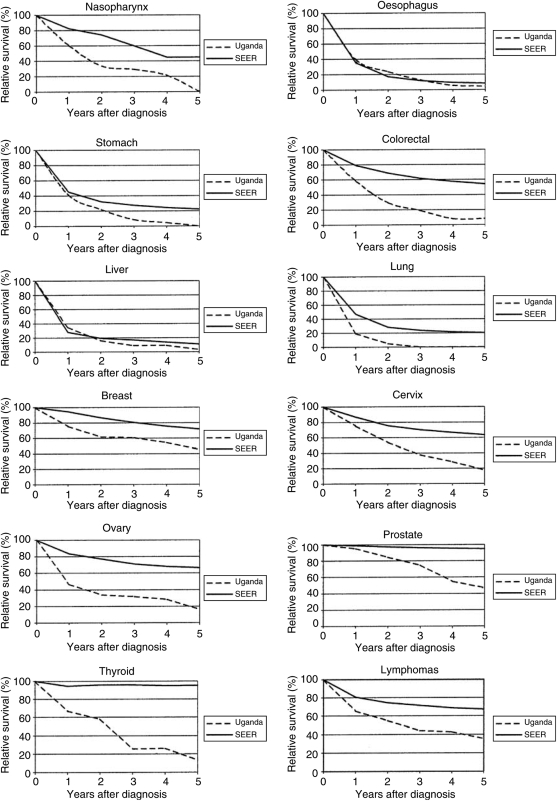
5-year relative survival (in %) of cancer patients in Kampala, Uganda and in the United States (SEER, Black Americans), 1993–1997.

Figure 1 shows the 5-year relative survival curves of the Ugandan patients compared to the age-adjusted curves of black American patients. The most dramatic difference was seen among patients with thyroid cancer, who can be cured today in the United States in almost every case: 95% of the patients survived the first year after diagnosis, after which no excess mortality due to the cancer was experienced. In contrast, Ugandan patients experienced excess mortality over the entire follow-up period, and the relative survival was 12% 5 years after the diagnosis. In general, the relative survival curves of patients with small chances of cure even in developed countries, such as oesophageal, stomach, liver, and lung cancer, are very similar in the two patient groups. Patients with cancers that have more promising treatment regimens or where screening and early diagnosis is possible have much better survival in the United States. In particular, very large survival disadvantages of Ugandan patients were apparent for nasopharyngeal, colorectal, cervical, ovarian, and prostate cancer.

## DISCUSSION

The prognosis of patients in this Ugandan study population was very poor, and much lower for almost all forms of cancer than the prognosis of patients in the United States or other developed countries. These survival figures are even lower than most previously published survival estimates from other developing countries (Sankaranarayanan *et al*, 1998). In particular, Ugandan patients with nasopharyngeal, colorectal, ovarian, and thyroid cancer experience much lower survival than patients in other, non-African developing countries. The very low survival estimates for patients with oesophageal, stomach, liver, and lung cancer are similar to those observed in other developing countries, but survival from these cancers is generally poor all over the world. The survival of patients with breast or prostate cancer and lymphomas was similar to that observed in other developing countries.

Although shocking, these survival estimates are unfortunately not surprising. Uganda is a very poor country: annual per capita health expenditure is currently estimated at only 36 dollars (WHOSIS, 2000), and 40% of the population live in absolute poverty (living on less than 1$ a day and unable to afford enough food to consume 2000–3000 calories a day) (Kikule, 2003). Given the constraints posed by the limited resources, the availability of cancer care may depend on the patient's financial contribution. At the same time, there is a lack of resources at every level of cancer care. In 1998, there were only two radiotherapy units and one chemotherapy unit in the country, and only an estimated 5% of patients had access to these facilities. Further constraints are posed by the lack of medical personnel: in Kampala, there are only approximately 50 doctors per 100 000 people (USA: 279) (WHOSIS, 1998; Merriman, 1999; Merriman and Heller, 2002). In 1993, a hospice service was started in Kampala to provide mainly home based palliative care for cancer and AIDS patients. The hospice services, which are never refused if a patient is unable to contribute to the cost of the service (Merriman and Heller, 2002), are probably the most widely utilised cancer services in the study area. The hospice service, which provided 20% of the vital status information in this study, is a great success and functions as a model for other African countries wishing to set up a patient-oriented hospice service (Merriman and Heller, 2002).

Completeness and accuracy of the data are always of concern in registry studies of survival, and particularly so in those from developing countries (Sankaranarayanan *et al*, 1998). In Kampala, the collection of follow-up data was particularly challenging and difficult, as described in detail in the methods section. As a result, a large proportion of patients could not be included or were lost to follow-up before study closure. This gives rise to concern that the survival estimates may be biased. However, although the vital status of lost-to-follow-up patients is unknown, the study personnel managed on many occasions to acquire some information about these patients from different sources, such as neighbours, landlords, etc. According to these notes, many of them went ‘up-country’, most probably back to their own community. The fact that these patients were not recorded at any health institution means that they are most probably not getting any treatment because specialised treatment is not available outside Kampala. Furthermore, it has been documented that in the Ugandan context patients prefer to be at home and be cared for by their families when they are ill. Patients are usually discharged from the hospital when they can not be helped any more, and they prefer to go home as people are customarily buried in their family village when they die (Kikule, 2003). It is therefore unlikely that patients who were lost-to-follow-up had had a much higher survival than patients with completed follow-up. Furthermore, the effect of loss to follow-up on population-based survival estimates in developing county settings has been previously assessed and was found to be marginal, even when lost to follow-up proportions were high (30–40%) and nonrandom with regard to prognostic factors (Swaminathan *et al*, 2002; Sriamporn *et al*, 2004). Therefore, it is reasonable to suppose that despite considerable exclusion and lost to follow-up, our results are free from a meaningful bias and adequately reflect the true survival experience of cancer patients in Uganda.

Stage is often considered to be the most important factor determining survival. Unfortunately, only sporadic information was available on this factor in our data set. However, it is commonly believed that stage at diagnosis is advanced in Africa for many cancers (Parkin *et al*, 2003), and as screening and education programmes are almost nonexistent, there is little reason to believe that this observation is not correct. Given that more than 60% of deaths occurred within the first year after diagnosis, and about 80% of deaths occurred within the first 2 years after diagnosis in this study population, it seems reasonable to assume that many of these patients were only diagnosed very late, when effective treatment is rarely possible.

In conclusion, the survival of cancer patients in Uganda is very poor and in its totality worse than that was reported from other non-African developing countries. Despite the challenging context and the discussed limitations, we are convinced that the data collected and the results are consistent with the described health care realities and accurately reflect the state of cancer management in Uganda today. Cancer management faces an enormous task in the country, and should be focused on those cancers, which pose the largest burden on the population, that may be prevented, or screened for comparatively easily, and for which deliverable treatment promises with positive outcome.

On the policy level, the results underscore the importance of the consistent application of the national cancer control programme guidelines as outlined by the WHO (WHO, 2002). In addition, ongoing efforts should be continued to increase the availability of the very successful hospice services, particularly in the rural areas of the country.

## Figures and Tables

**Table 1:** Cancer patients included in the survival analysis in Kampala, Uganda, 1993–1997

**Cancer site**	**No.^a^**	**Patients with complete data**	**Patients lost to follow-up (%)**	**Histologically verified (%)**
Nasopharynx	50	31	19 (30.2)	78.0
Oesophagus	182	151	31 (15.8)	39.6
Stomach	91	67	24 (23.1)	48.4
Colorectal	104	81	23 (20.0)	60.6
Liver	117	101	16 (12.0)	33.3
Lung	50	41	9 (16.4)	62.0
Breast	174	96	78 (33.5)	63.2
Cervix uteri	285	149	136 (29.6)	63.9
Ovary	69	48	21 (28.0)	52.2
Prostate	161	119	42 (19.7)	72.7
Eye	88	39	49 (35.0)	72.7
Thyroid	41	20	21 (41.2)	80.5
Lymphomas	199	117	82 (32.7)	79.4
Kaposi sarcoma	220	145	75 (30.1)	83.2
				
Total	1831	1205	626 (26.8)	61.8

a Excluding patients with missing/invalid data or no follow-up after diagnosis.

**Table 2:** Median age of cancer patients in Kampala, Uganda, compared with patients from the United States, 1993–1997

**Cancer site**	**Uganda**	**USA Black American patients**
Nasopharynx	32	50
Oesophagus	58	63
Stomach	55	68
Colorectal	53.5	67
Liver	42	62
Lung	48	65
Breast	45	56
Cervix	43	49
Ovary	40	61
Prostate	70	68
Eye	28	2
Thyroid	46	45
Lymphomas	10	46
Kaposi Sarcoma HIV+ (*N*=188)	30	—
Kaposi Sarcoma HIV− (*N*=32)	29	

**Table 3:** Absolute and relative survival (in %) of Ugandan patients with cancer, Kampala, Uganda, 1993–1997

**Cancer site**	**5-year absolute survival**	**s.e.^a^**	**5-year relative survival**	**s.e.**
Nasopharynx	0.0	—	0.0	—
Oesophagus	3.5	1.8	4.5	2.3
Stomach	0.0	—	0.0	—
Colorectal	6.6	3.1	8.3	3.9
Liver	2.7	1.9	3.2	2.2
Lung	0.0	—	0.0	—
Breast	38.4	6.0	45.4	7.1
Cervix	15.9	4.2	18.2	4.8
Ovary	14.1	7.0	16.2	8.1
Prostate	29.5	4.8	46.9	7.7
Eye	31.3	9.0	34.2	9.8
Thyroid	11.8	10.0	13.4	11.4
Lymphomas	33.4	5.5	35.4	5.8
Kaposi sarcoma HIV+	8.2	3.2	9.1	3.6
Kaposi sarcoma HIV−	60.1	13.0	65.7	14.2

a s.e.: standard error.

## References

[bib1] Brenner H, Hakulinen T (2003) On crude and age-adjusted relative survival rates. J Clin Epidemiol 56: 1185–1191 14680669 10.1016/s0895-4356(03)00209-9

[bib2] Brenner H, Hakulinen T, Gefeller O (2002) Computational realization of period analysis for monitoring cancer patient survival. Epidemiology 13: 611–612 12192239 10.1097/00001648-200209000-00031

[bib3] Chokunonga E, Ramanakumar AV, Nyakabau AM, Borok MZ, Chirenje ZM, Sankila R, Parkin DM (2004) Survival of cervix cancer patients in Harare, Zimbabwe, 1995–97. Int J Cancer 109: 274–277 14750180 10.1002/ijc.11670

[bib4] Ederer F, Axtell LM, Cutler SJ (1961) The relative survival rate: a statistical methodology. Natl. Cancer Inst Monogr 6: 101–121 13889176

[bib5] Gondos A, Chokunonga E, Brenner H, Parkin DM, Sankila R, Borok MZ, Chirenje ZM, Nyakabau AM, Bassett MT (2004) Cancer survival in a southern African urban population. Int J Cancer 112: 860–864 15386382 10.1002/ijc.20471

[bib6] Greenwood M (1926) A report on the natural duration of cancer. Ministry of Health, His Majesty's Statistical Office, London

[bib7] Hakulinen T (1982) Cancer survival corrected for heterogeneity in patient withdrawal. Biometrics 38: 933–942 7168796

[bib8] Kikule E (2003) A good death in Uganda: survey of needs for palliative care for terminally ill people in urban areas. BMJ 327: 192–194 12881259 10.1136/bmj.327.7408.192 PMC166119

[bib9] Lopez AD, Ahmad OB, Guillot M, Inoue M, Ferguson BD, Salomon JA (2001) Life tables for 191 countries for 2000: Data, methods, results (GPE discussion paper No. 40). In: Health systems performance assessments peer review, technical documentation. IV outcomes: population health. Evidence and information for policy (EIP), WHO

[bib10] Merriman A (1999) Hospice Uganda: 1993–1998. J Pall Care 15: 50–52 10333665

[bib11] Merriman A, Heller KS (2002) Hospice Uganda – A model palliative care initiative in Africa: an interview with Anne Merriman. Innovations in End-of-Life Care; 4, Available from: http://www2.edc.org/lastacts/archives/archivesMay02/intlpersp.asp, (web-based journal)

[bib12] Parkin DM, Ferlay J, Hamdi-Chérif M, Sitas F, Thomas J, Wabinga H, Whelan S (eds) (2003) Cancer in Africa: Epidemiology and Prevention. Lyons: IARC Sci Publ No. 153. IARC

[bib13] Parkin DM, Wabinga H, Nambooze S (2001) Completeness in an African cancer registry. Cancer Causes Control 12: 147–152 11246843 10.1023/a:1008966225984

[bib14] Parkin DM, Wabinga H, Namboozes S, Wabwire-Mangen F (1999) AIDS related cancers in Africa: maturation of the epidemic in Uganda. AIDS 13: 2563–2570 10630526 10.1097/00002030-199912240-00010

[bib15] Sankaranarayanan R, Black RJ, Parkin DM (eds) (1998) Cancer Survival in Developing Countries. Lyons: IARC Sci Publ No. 145. IARC 10194635

[bib16] SEER*Stat 4,2 (2002) SEER cancer incidence public-use database, 1973–1999, November 2001 Submission, Issued April 2002

[bib17] Sriamporn S, Swaminathan R, Parkin DM, Kamsa-ard S, Hakama M (2004) Loss-adjusted survival of cervix cancer in Khon Kaen, Northeast Thailand. Br J Cancer 91: 106–110 15199396 10.1038/sj.bjc.6601959 PMC2364753

[bib18] Swaminathan R, Sankaranarayanan R, Hakama M, Shanta V (2002) Effect of loss to follow-up on population based cancer survival rates in developing countries. Int J Cancer 100(Suppl 13): 172 (18th UICC Cancer Congress, 30 June–5 July 2002, Oslo, Norway – Abstract book)12115566

[bib19] Wabinga HR, Parkin DM, Wabwire-Mangen F, Mugerwa JW (1993) Cancer in Kampala, Uganda, in 1989–91: changes in the era of AIDS. Int J Cancer 54: 26–36 8478145 10.1002/ijc.2910540106

[bib20] Wabinga HR, Parkin DM, Wabwire-Mangen F, Nambooze S (2000) Trends in cancer incidence in Kyadondo County, Uganda, 1960–1997. Br J Cancer 82: 1585–1592 10789729 10.1054/bjoc.1999.1071 PMC2363394

[bib21] Wabinga H, Ramanakumar AV, Banura C, Luwaga A, Nambooze S, Parkin DM (2003) Survival of cervix cancer patients in Kampala, Uganda, 1995–1997. Br J Cancer 89: 65–69 12838301 10.1038/sj.bjc.6601034 PMC2394214

[bib22] WHO National Cancer Control Programmes (2002) Policies and Managerial Guidelines 2nd edn Geneva: World Health Organization http://www.who.int/entity/cancer/media/en/408.pdf

[bib23] WHO Statistical Information System (WHOSIS) (around 1998) WHO estimates of health personnel. Physicians, nurses, midwives, dentists and pharmacists. Available from: http://www3.who.int/whosis/health_personnel/health_personnel.cfm

[bib24] WHO Statistical Information System (WHOSIS) (2000) Comparison on selected indicator within WHO Region. Uganda (Compared with other countries in WHO African Region): Indicator: Per capita total expenditure on health in international dollars, http://www3.who.int/whosis/country/compare.cfm?country=uga&indicator=strPcTotEOHinIntD2000&language=english

[bib25] Ziegler JL, Newton R, Katongole-Mbidde E, Mbulataiye S, De Cock K, Wabinga H, Mugerwa J, Katabira E, Jaffe H, Parkin DM, Reeves G, Weiss R, Beral V (1997) Risk factors for Kaposi's sarcoma in HIV-positive subjects in Uganda. AIDS 11: 1619–1626 9365767 10.1097/00002030-199713000-00011

